# Synergistic Antibacterial Effect of Mucus Fraction from *Cornu aspersum* and Cirpofloxacin Against Pathogenic Bacteria Isolated from Wounds of Diabetic Patients

**DOI:** 10.3390/antibiotics14030260

**Published:** 2025-03-04

**Authors:** Mila Dobromirova Kaleva, Momchil Kermedchiev, Lyudmila Velkova, Maya Margaritova Zaharieva, Aleksandar Dolashki, Maria Todorova, Maya Guncheva, Pavlina Dolashka, Hristo Miladinov Najdenski

**Affiliations:** 1 Department of Infectious Microbiology, The Stephan Angeloff Institute of Microbiology, Bulgarian Academy of Sciences, 26 Acad. G. Bonchev Str., 1113 Sofia, Bulgaria; mdkaleva@microbio.bas.bg (M.D.K.); zaharieva26@yahoo.com (M.M.Z.); 2 Institute of Organic Chemistry with Centre of Phytochemistry, Bulgarian Academy of Sciences, Acad. G. Bonchev Str., bl. 9, 1113 Sofia, Bulgaria; kermedchiew@yahoo.com (M.K.); lyudmila.velkova@orgchm.bas.bg (L.V.); aleksandar.dolashki@orgchm.bas.bg (A.D.); krasimirova_m@yahoo.com (M.T.); maya.guncheva@orgchm.bas.bg (M.G.); or pda54@abv.bg (P.D.); 3 Centre of Competence “Clean Technologies for Sustainable Environment—Waters, Waste, Energy for a Circular Economy”, 1000 Sofia, Bulgaria

**Keywords:** snail mucus from *Cornu aspersum*, diabetic foot ulcers, multidrug resistant bacteria, ciprofloxacin, synergistic combinations

## Abstract

**Background/Objectives**: The treatment of diabetic foot ulcers (DFU) is a challenging medical problem of extreme clinical and social importance, as a consequence of the emerging antibiotic resistance and decreased quality of life of diabetic patients due to impaired wound healing. One of the current trends in world science is the search for biologically active substances derived from living organisms. Biologically active peptides from snail mucus attract considerable scientific interest because of their pleiotropic pharmacological properties. The aim of our study was to evaluate the activity of a combination between a snail mucus protein fraction (MW > 20 kDa) obtained from the garden snail *Cornu aspersum* and the clinically applied antibacterial chemotherapeutic ciprofloxacin on pathogenic bacterial strains isolated from DFU. **Results**: The test bacterial strains were characterized as multidrug resistant. The combination between ciprofloxacin and the snail mucus fraction of interest led to additive or synergistic effects depending on the test strain. The mucus fraction exerted a well-pronounced wound-healing effect and no cytotoxicity on normal human fibroblasts and keratinocytes. **Methods**: The snail mucus was obtained by a patented technology (BG Utility model 2097/2015) and its electrophoretic profile was presented by SDS-PAGE. The bacterial strains were identified and tested for antimicrobial susceptibility (BD Phoenix M50 and Kirby–Bauer assay). The in vitro cytotoxicity of the mucus was evaluated by ISO 10995-5. The antimicrobial activity and combination effects were tested through ISO 20776/1 and the Checkerboard assay. **Conclusions**: The obtained results are promising and open new horizons for the development of novel combination treatment schemas for healing of infected DFU.

## 1. Introduction

Biologically active substances extracted from living organisms are widely used in various biotechnological processes in the pharmaceutical, cosmetic, food and beverage, etc., industries [1]. Snail mucus is rich in biologically active substances with pleiotropic pharmacological properties, ranging from protective and antimicrobial to immunostimulating, wound healing, anti-inflammatory, etc., which reveals their significant potential for clinical application [2,3]. Modern trends in science are aimed at in-depth study of the complex components of snail mucus secretions with the aim of isolating new bioactive molecules with various applications in medical practice [3].

It is known that the mucus secretion of land snails is a multicomponent biosubstance containing a number of bioactive compounds with different molecular weights and pharmacological properties such as antimicrobial, anti-inflammation; antioxidant and regenerative [3,4,5,6,7,8,9,10,11]. In general, the mucus secretion of snail and slug contains 90–99.7% water *w*/*w*, the remaining 0.3–10% mucus is known to include metal ions, secondary metabolites (amino acids, allantoin, glycolic acid, etc.), antimicrobial peptides (AMPs), proteins and glycoproteins, hyaluronic acid, and glycosaminoglycans [3]. According to scientific data, including our own preliminary studies, it has been proven that the antimicrobial activity of the mucus of land snails is due to the presence of AMPs, certain proteins and glycoproteins, which are characterized by antimicrobial potential [10,12,13,14]. The mucus of a number of land snail species (*Achatina fulica*, *Helix aspersa*, *Cryptozona bistrialis*, *Lissachatina fulica*, *Hemiplecta differenta* and *Cornu aspersum*) contains proteins and glycoproteins with antimicrobial properties [10,12,13,15,16,17,18,19].

Components in gastropod mucus are known to accelerate cellular processes associated with tissue regeneration, making them suitable for certain medical applications, such as wound healing, especially in the presence of concomitant infection [2]. Several studies have reported the healing potential of the mucus of the land snails *Achatina fulica, Archachatina marginata* and *Helix lucorum* in wound healing [20,21]. Recently Nuryana et al. (2020), founded that *A. fulica* mucous improves cell viability of UVB-irradiated human fibroblast culture which associates with increases collagen deposition and reducing collagen I/collagen III ratio [22]. Several studies have shown that the mucilage of *Cornu aspersa* also accelerates wound healing and exhibits antioxidant properties [5,23,24,25].

Furthermore, the presence of antimicrobial peptides and proteins within the mucus opens possibilities for novel antimicrobial agents, particularly relevant in the current era of emerging antibiotic resistance [7,10]. In recent years, the strategy to use different antimicrobial agents in combination schemas to achieve synergistic effects has been under investigation in the fight against microbial resistance [26,27]. This approach represents an attractive therapeutic modality because it opens a possibility to target non-overlapping signaling pathways and decreases the risk of developing cross-resistance [28]. Appropriate combinations of different antimicrobial therapeutics lead to dose reduction, as well as reduced toxicity, fewer side effects, better synergistic effect, attack of multiple target sites, reduced risk, and manifestation of potent and rapid antibacterial effects against multidrug-resistant pathogens [29].

Diabetes is a socially significant disease with multiple complications affecting the quality of life of the patients. Hyperglycemic conditions in these patients cause peripheral neuropathies and circulatory disorders due to vascular damage which leads to the development of the so-called diabetic ulcers (DU) mainly on the lower extremities. Data indicate that worldwide, diabetic foot ulcers (DFU) affect about 18.6 million people annually, leading to frequent amputations and death [30]. Frykberg et al. (2020) and Gong et al. (2019) indicate that every 20th diabetic patient is admitted to hospital for amputation of a lower limb as a result of DFU [31,32]. The wound areas are characterized by ischemia and hypoxia. In these stagnant processes, all stages of wound healing are affected—hemostasis, the phases of inflammation, proliferation and reconstruction are prolonged [33,34,35,36,37,38]. An essential factor for the development of DFU is the huge amount of proliferating macrophages at the site of injury for a prolonged period of time [39]. Therefore, impaired wound healing is a common complication in patients with diabetes and successful treatment of DFU is of extreme clinical and social importance [33,34,35,36,37]. Due to the compromised immune status of the macro-organism by diabetes and the frequent use of antibiotics in the society required for treatment of recurrent microbial infections, the increasing of multidrug-resistant bacteria is an emerging medical problem [40]. DFU occurs when there is an abscess in the wound and the adjacent tissues are inflamed. Bacterial infection of the wound surface is related also to biofilm formation which prevents healing of the wound site, causes additional inflammation of the surrounding tissues and s the removal of bacteria by antibiotics [41].

The most commonly isolated bacteria from DFU are *Staphylococcus aureus* and other Gram-positive cocci, *Pseudomonas* spp., *Escherichia coli*, *Morganella morganii*, *Proteus mirabilis*, *Acinetobacter* spp. and *Enterococcus* spp. [42,43,44,45,46,47,48,49,50]. Bacteria from the genera *Klebsiella* sp. and *Bacillus* sp. are also part of the polybacterial composition of biofilms on DFU [51].

Considering the current state of the art, in the present study we aimed at investigating the synergistic effect of a combination between protein mucus fraction with molecular weight above 20 kDa (MW > 20 kDa) isolated from the garden snail species *Cornu aspersum* and the fluoroquinolone ciprofloxacin (CIP) which is an antibacterial chemotherapeutic with a broad antibacterial spectrum and wide clinical application. As an in vitro model for studying the desired antibacterial activity of the combination, we used bacterial isolates from DFU identified as *Staphylococcus aureus*, *Enterococcus faecalis* and *Pseudomonas aeruginosa* and characterized for antimicrobial resistance against a panel of clinically applied antibiotics. In addition, an evaluation of the in vitro proliferation activity of the mucus fraction was performed on non-tumorigenic human fibroblasts and keratinocytes in order to show the potential for a regenerative effect and the absence of cytotoxicity.

## 2. Results

### 2.1. Preparation and Characterization of Mucus Fraction with Molecular Weight Above 20 kDa

The raw mucus was collected from garden snails *C. aspersum*, grown on Bulgarian farms by a patented technology using a low-voltage electrical stimulation device that does not damage the biological functions of the snails, according to Bulgarian utility model 2097/2015 [52]. The preparation of a standardized purified mucus extract (also subject to patent protection of the above-mentioned utility model) is achieved after several filtration cycles at 4 °C of the mucus supernatant after removal of coarse impurities from the crude mucus extract by centrifugation [6,10,52]. The target protein fraction containing compounds with a MW > 20 kDa was produced by pressure ultrafiltration of purified total mucus extract on a polyethersulfone membrane with a 20 kDa pore size (Microdyn Nadir™ from STER-LITECH Corporation, Goleta, CA, USA, respectively) using an Amicon^®^ Stirred Cell 200 mL (UFSC20001, MerckMillipore, Merck Group, Darmstadt, Germany) connected to an external source of gas (N_2_) [6,10]. The application of this non-invasive method ensures the production of fractions containing intact compounds. Using the Bradford assay, the concentration of the target mucus fraction with MW > 20 kDa was determined to be 2.50 mg/mL protein.

**Figure 1:**
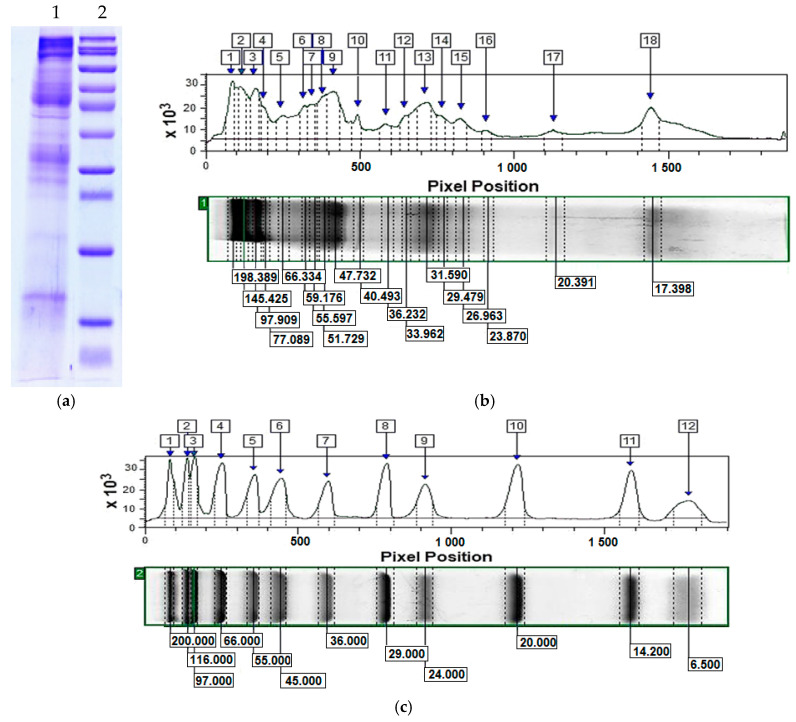
Electophoretical analysis of target fraction with MW > 20 kDa from *C. aspersum* mucus by 12% SDS-PAGE and ImageQuant^TM^ TL v8.2.0 software. Legend: (**a**) lane 1—protein fraction from *C. aspersum* mucus with an MW > 20 kDa and lane 2—standard protein marker with a molecular weights between 200.0 and 6.5 kDa (SigmaMarker^TM^, Sigma-Aldrich, Saint Louis, MO, USA); (**b**) the electrophoretic profile analyzed by ImageQuant^TM^ TL of target mucus fraction on an electrophoretic lane 1; (**c**) the electrophoretic profile of a standard protein marker (electrophoretic line 2).

The electrophoretic profile of the mucus fraction with an MW > 20 kDa from *C. aspersum* was analyzed at a concentration of 1.25 mg/mL by 12% SDS-PAGE. The result shows various protein bands with MWs mainly between 20 and 200 kDa (Figure 1a). The determined protein profile of the mucus fraction with an MW > 20 kDa (Figure 1b) is in line with the results reported in [10], which confirms the good reproducibility of the method for snail mucus collection and the target protein mucus fraction, as well as the stability of the content of different consecutively collected snail mucus batches.

### 2.2. Evaluation of Viability of Skin Fibroblasts (BJ) and Human Keratinocytes (HaCaT) Treated by Different Mucus Fractions

**Figure 2:**
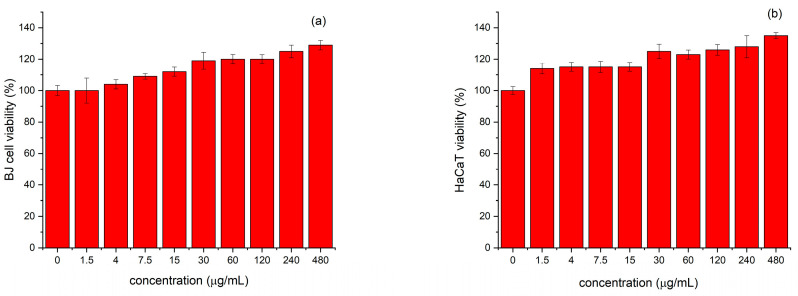
Viability of non-tumorigenic human cell lines treated with a mucus fraction with MW > 20 kDa from *Cornu aspersum* for 24 h. Legend: (**a**) human skin fibroblasts (BJ) and (**b**) human keratinocytes (HaCaT); control sample—untreated cells (100% viability).

In order to evaluate the cytotoxicity, the effect of the mucus fraction with MW > 20 kDa was investigated on the viability of non-tumorigenic skin fibroblasts (BJ) and human keratinocytes (HaCaT) and was evaluated in a wide concentration range from 1.5 to 480.0 µg/mL for 24 and 48 h. The viability of the non-treated cells (control) for the same incubation period was taken as 100%. The results obtained after 24 h of exposure are shown in Figure 2. The comparison between 24 and 48 h of treatment did not lead to any differences. As can be seen, the mucus fraction did not show cytotoxicity in the concentration range tested. Moreover, it even stimulated the proliferation of BJ and HaCaT cells in a concentration dependent manner. In difference to the non-treated control cells, we observed an increase up to 20% or higher in the population of BJ cells treated with 120 µg/mL or higher concentration of the tested mucus fraction as compared to the untreated control. Similar induction of the cell proliferation was observed also by the HaCaT cell line.

### 2.3. In Vitro Evaluation of the Wound-Healing Capacity of Cornu aspersum Mucus Protein Fraction with MW > 20 kDa

**Figure 3:**
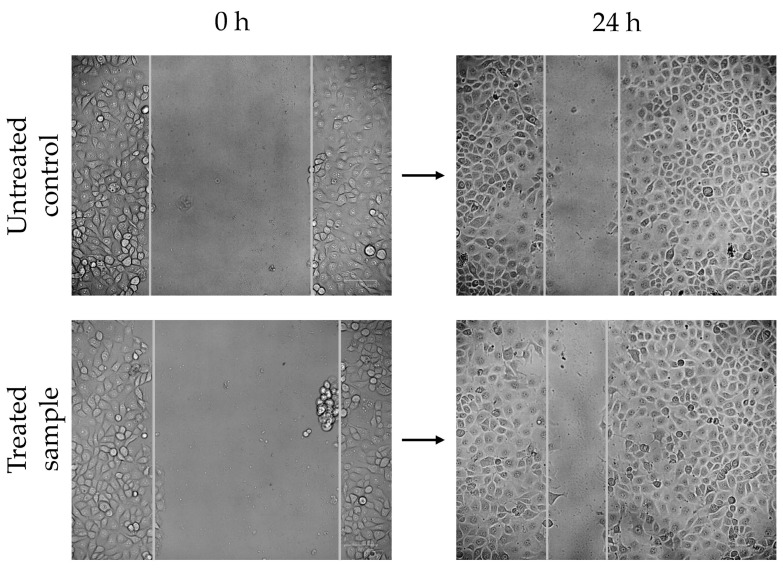
In vitro “wound-healing” assay on HaCaT cells treated with mucus fraction with MW > 20 kDa in comparison to the untreated control cells. Photographic images were taken on a Zoe Fluorescent Cell Imager (Bio-Rad).

The wound-healing capacity of the *C. aspersum* mucus protein fraction on HaCaT cells was evaluated by a scratch test. Figure 3 shows images taken immediately after wound formation and 24 h after treatment with the test mucus fraction. The results showed that the fraction containing proteins with MW > 20 kDa accelerated the migration and proliferation of HaCaT cells.

### 2.4. Morphological and Biochemical Characteristic of Pathogenic Bacterial Isolates from Patients with Diabetes

Four bacterial isolates from DFU of two patients were confirmed with the BD Phoenix M50 automated system: *Staphylococcus aureus* and *Pseudomonas aeruginosa* from sample 1 and *Enterococcus faecalis* and *P. aeruginosa* from sample 2. The results from the biochemical identification are presented in Table 1.

### 2.5. Antimicrobial Susceptibility (AMR) of Pathogenic Bacterial Isolates from Patients with Diabetes

The antibiotic resistance of the clinical isolates was determined by the Kirby–Bauer disk diffusion method based on their inhibition zones and with the automated microbiology system Phoenix BD M50 based on the MICs of the antibiotics/chemotherapeutics.

The Kirby–Bauer test revealed resistance of the four bacterial isolates to the following clinically applied antibiotics: (1) amoxicillin/clavulanic acid, ampicillin, ceftriaxone, ciprofloxacin, chloramphenicol, clarithromycin, tetracycline, erythromycin, gentamicin, penicillin, vancomycin, rifampin for *S. aureus* and *E. faecalis*, and (2) amikacin, amoxicillin/clavulanic acid, ampicillin, ceftriaxone, ciprofloxacin, gentamicin, meropenem, norfloxacin, oxacillin, trimethoprim/sulfamethoxazole for *P. aeruginosa*. The results for the Gram-positive isolates are summarized in Table 2.

The results from the Kirby–Bauer test performed with the Gram-negative isolates belonging to the species *P. aeruginosa* are presented in Table 3.

The resistance pattern of the four isolates determined by the BD Phoenix™ M50 automated microbiology system directly after the identification in the same BD Phoenix™ panels can be summarized as follows: (1) the *S. aureus* isolate was resistant to the antibiotics ampicillin, cefoxitin, erythromycin, and gentamicin; (2) the *Enterococcus faecalis* isolate showed resistance towards the antibiotics cefoxitin, ceftaroline, clindamycin, erythromycin, and gentamicin; (3) both *Pseudomonas aeruginosa* isolates were not susceptible to the antibiotics ceftazidin, cefuroxime, imipenem and the antimicrobial chemotherapeutic ciprofloxacin. The AST performed with the automated systems completes and confirms the data from the Kirby–Bauer test.

### 2.6. Minimal Inhibitory Concentrations After Single Administration and Combination Effects

The minimal inhibitory concentrations of the snail mucus fraction with MW > 20 kDa and CIP after single application, as well as the combination effects are presented in Table 4. Single treatment with snail mucus did not visibly inhibit the bacterial growth of all clinical isolates as determined by the BMD test. The MICs of the chemotherapeutic CIP inhibited the growth of all isolates in concentrations below the clinical breakpoints for the relevant species. The combination between both led to an effect which is additive to synergistic or synergistic effect in all four isolates as the snail mucus diminishes the effective concentration of CIP two-fold. As the mucus fraction was not active at the highest concentration possible for administration, a four-fold higher concentration of 1018 mg/L was hypothetically used in the calculations regarding the *E. faecais* and both *P. aeruginosa* isolates.

## 3. Discussion

In the current study, for the first time we investigated the synergistic effect between a protein fraction with a MW of over 20 kDa from the *C. aspersum* mucus and the fluoroquinolone ciprofloxacin against multidrug-resistant pathogenic bacterial isolates from two patients with chronic DFU. Our choice of the protein fraction with a MW > 20 kDa from the mucus is based on our previous results revealing its promising antibacterial activity against five pathogenic and conditionally pathogenic bacterial strains (*B. cereus* 1085, *P. acnes* 1897, *S. enterica* 8691, *E. faecalis* 3915, and *E. faecium* 8754) compared to vancomycin [10]. The performed proteomic analysis of this fraction visualized a number of proteins characterized by a high homology with proteins known for their potential antibacterial activity such as the mucus protein aspernin, hemocyanins, H-lectins, L-amino acid oxidase-like protein and mucins (mucin-5AC, mucin-5B, mucin-2 and mucin-17). Furthermore, the same mucus components show high homology with compounds with regenerative properties such as a elastin-like (XP_056017499.1), collagen alpha-1(IV) chain-like (XP_025114158.1), collagen α-4(VI) chain-like (XP_059154404.1); collagen α-1(XII) chain-like (XP_059154404.1) [10]. The electrophoretic profile of the fraction used (Figure 1) in the present study is in full accordance with our previously published data [10].

Regarding the antibacterial activity, mucus fraction with MW > 20 kDa was tested on four pathogenic bacterial strains, isolated from patients with DFU. Single application of the mucus fraction with MW > 20 kDa did not lead to the desired bactericidal effect up to concentration 225 µg/mL or higher depending on the pathogen species. The Gram-positive isolate *S. aureus* was more sensitive than the other Gram-positive isolate *E. faecalis* or the Gram-negative isolates belonging to the species *P. aeruginosa*. As previously reported by the authors, snail mucus fraction > 20 kDa possesses strong antibacterial activity against sensitive strains from the genera *Bacillus*, *Salmonella* and *Enterococcus* in concentrations in the range of 32–128 µg/mL [10]. The tested bacterial isolates in this study were characterized by a resistance towards a number of clinically applied antibiotics and chemotherapeutics which can explain the lower activity of the mucus fraction by single application. The *P. aeruginosa* isolates were resistant or intermediate resistant towards ampicillin, amoxicillin/clavulanic acid, meropenem, and the fluoroquinolones CIP, norfloxacin and ofloxacin, as well as DNA synthesis inhibitor trimethoprim/sulfametoxazol. The listed antimicrobial agents belong to four different groups of antibiotics with different mechanisms of action, which points to a multidrug resistance. The *S. aureus* isolate was resistant also to four antibiotics, belonging to different pharmacological subgroups of the inhibitors of the cell wall synthesis (ampicillin and cefoxitin) and the inhibitors of the protein synthesis (macrolides and gentamicin). The *E. faecalis* isolate showed resistance to five antibiotics—cefoxitin, ceftaroline, clindamycin, erythromycin, and gentamicin. The *P. aeruginosa* isolates were resistant to ceftazidin, cefuroxime, imipenem and 3 fluoroquinolones (ofloxacin, norfloxacin and ciprofloxacin). Given that fact, we chose CIP as an appropriate agent for combination with the mucus protein fraction aiming at enhancing the drug activity.

The combination between the mucus fraction and the clinically applied fluoroquinolone ciprofloxacin led to additive or synergistic effects depending on the type of the isolate. Very low concentrations of the mucus fraction in the combination (4 µg/mL) were enough to enhance the activity of CIP two-fold (Table 4). Antibiotics are part of the therapeutic schema of DFU [53,54]. Developing of resistance mechanisms may impede their effect at active concentration [55]. The presence of a synergistic effect is indicative for a different mode of action of the *C. aspersum* mucus and that of ciprofloxacin which is a gyrase inhibitor [56,57]. Our results are in line with other studies where authors investigated and proved the antibacterial potential of mucus obtained from African giant snail *Achatina fulica Férussac* [58] and other species such as *Archachatina marginata*, *Achatina achatina*, and *Achatina fulica* [12,16,59]. El-Zawawy et al. investigated antimicrobial efficacy of Egyptian *Eremina desertorum* and *H. aspersa* snail mucus and found out that they possess an inhibitory effect on bacterial and fungal strains in the concentration range 5–32 µg/mL, whereby the antibacterial activity was stronger than the antifungal [60]. The authors also proved the anti-inflammatory and wound repairing potential of both mucus fractions. The study of Alarfaj et al. also confirms the antibacterial potential of the snail mucus, whereby the active antibacterial concentrations were higher than the reported from the other cited publications and are between 25 and 100% as proven by agar diffusion assay [60]. As to our knowledge, this is the first study on the synergistic activity of a *C. aspersum* mucus fraction with the fluoroquinolone ciprofloxacin; therefore, further investigations are needed in order to elucidate the exact mechanism of action of the combination.

Evaluation of the cytotoxicity of the fraction with MW > 20 kDa on skin fibroblasts (BJ) and human keratinocytes (HaCaT) clearly showed not only a lack of cytopathic effect, but on the contrary—stimulation of the proliferation of both cell lines in a concentration-dependent manner. The observed stimulation effect of the tested protein mucus fraction with MW > 20 kDa on the proliferation of BJ and HaCaT is consistent with studies of other authors on the cytotoxicity of mucus isolated from other species, e.g., the slugs *Limax maximus* and *Arion rufus* and snail *Helix aspersa* Muller (HelixComplex) [1,23]. Leskow et al. (2021) showed that mucus extracts from *L. maximus* and *A. rufus* are tolerated by keratinocytes in a broad concentration range (up to 1000 µg/mL). The mucus of slugs *L. maximus* and *A. rufus* improves the survival of keratinocytes and reduces the number of melanoma cells by up to 22%. *L. maximus* mucus showed a stronger influence on cell survival, while *A. rufus* mucus was characterized by higher cytotoxicity on both tested cell lines [1]. The study by Trapella et al. (2018) also established a lack of cytotoxicity of HelixComplex and found significant increase in cell number of non-tumorigenic mammalian fibroblasts (MRC-5 and NIH-3T3), treated with a concentration of 400 µg/mL at 48 and 72 h [23], a result which is comparable to our data for BJ cells treated with the tested mucus protein fraction (MW > 20 kDa) of *C. aspersum*.

Based on the identified proteins in the mucus fraction with MW 20 kDa and the obtained results for stimulation of BJ and HaCaT cells, we hypothesized that this protein mucus fraction would positively influence the wound healing process. The first step to prove this hypothesis was the in vitro scratch tests performed to evaluate the potential of the mucus fraction for wound healing (Figure 3). The obtained results showed a faster closure of the scratch after treatment with the protein fraction as compared to the untreated control. Our results are in accordance with the data presented by Trapella et al., 2018 [23]. The accelerated wound healing, resulting from increased migration and proliferation of HaCaT cells, is most probably due to the unique composition of the mucus fraction with MW > 20 kDa. Recently, proteins characterized by a high homology with the elastin-like protein; several types of collagen (collagen alpha-1, collagen α-4, and collagen alpha-6); and mucins (mucin-5AC-, mucin-5B-, mucin-2-, and mucin-17-like proteins), as well as enzymes with antioxidant activity, were identified as components of this fraction [10]. The study of Deng et al. (2023) demonstrated that the presence of sulfated glycosaminoglycan in the mucus of *A. fulica* and *H. lucorum* effectively promoted the healing of chronic wounds in a diabetic rat model by improving skin incision adhesion, as well as accelerating granulation tissue regeneration, angiogenesis and collagen deposition [13]. Mucins, which are an important component of the snail mucus, can play a key role for different biological functions, including adhesion, lubrication, and first line defense against bacterial infections [3,7]. Probably, the presence of mucins in the protein mucus fraction is one of the factors for the observed increase in cell migration of treated HaCaT cells.

Taken together, our data demonstrated a synergistic antibacterial effect between a protein mucus fraction with MW > 20 kDa and the antibacterial agent CIP on four pathogenic bacterial species isolated from DFU of diabetic patients. The combination inhibited more efficiently and by lower concentrations the growth of the pathogens as compared to single treatment. The mucus fraction itself exerted a significant wound-healing effect which could be beneficial for the tissue granulation during the proliferation phase of the healing process.

## 4. Materials and Methods

### 4.1. Snail Extract Preparation

The mucus was collected from garden snails *Cornu aspersum*, grown on Bulgarian farms using a patented technology created on the basis of low-voltage electrical stimulation without damaging the biological functions of the snails, as described previously in Bulgarian Utility Model 2097/2015 [52]. After removal of gross impurities (which are usually small particles of soil) from the crude mucus extract the supernatant mucus was purified by 3 cycles of filtration at 4 °C, with filters with smaller pore sizes being used for each subsequent filtration, which are also patentable [52].

The target fraction with MW above 20 kDa was obtained from the thus obtained total mucus extract by pressure ultrafiltration on membrane with pore sizes of 20 kDa (polyethersulfone, Microdyn Nadir™ from STER-LITECH Corporation, Goleta, CA, USA, respectively) using an Amicon^®^ Stirred Cell 200 mL (UFSC20001, MerckMillipore, Merck Group, Darmstadt, Germany) connected to an external source of gas (N_2_). The concentrations of the mucus fractions were determined by the Bradford assay [61].

### 4.2. Sodium Dodecyl Sulfate Polyacrylamide Gel Electrophoresis (SDS-PAGE) and Image Analysis by ImageQuant™ TL v8.2.0 Software

The electrophoretic analysis of the protein mucus fraction was performed by SDS-PAGE using 5% stacking gel and 12% resolving gel, according to the Laemmli method with modifications [62]. The following reagents were used: DL-dithiothreitol, acrylamide/bis-acrylamide (30% solution), bromophenol blue sodium salt (Sigma-Aldrich, Schnelldorf, Germany), N,N,N′,N′-tetramethylethylenediamine (TEMED), ammonium persulphate (APS) (GE Healthcare, Stockholm, Sweden), Laemmli sample buffer (2×), for SDS PAGE (SERVA, Heidelberg, Germany) and protein standard marker–mixture of proteins with molecular weights from 6.5 kDa to 200 kDa of SigmaMarker^TM^ (Sigma-Aldrich, Saint Louis, MO, USA).

After scanning, image analysis of an electrophoretic profile of the mucus fraction against the protein standard marker used was done by ImageQuant™ TL v8.2.0 software (GE Healthcare Bio-Sciences AB, Uppsala, Sweden) as previously described [10], according to [63].

### 4.3. Cell Lines and Culture Conditions

Two non-tumorigenic human cell lines were used for evaluation of the in vitro cytotoxicity and the proliferation effect of the tested mucus fraction (MW above 20 kDa). The BJ cell line (human skin fibroblasts) originates from the American Type Culture Collection (ATCC) and the HaCaT cell line (human keratinocytes) was purchased from the CLS GmbH (currently Cytion) situated in Eppelheim, Germany. Both cell lines were maintained according to the recommendations of the biobanks. The culture medium used consisted of Dulbecco modified Eagle medium high glucose with L-glutamine and sodium bicarbonate, Stable cell (DMEM), fetal bovine serum (FBS), and penicillin-Streptomycin solution (100×). The cells were sub-cultured with 0.25% trypsin–EDTA solution and sterile phosphate saline buffer, pH 7.4 (PBS) were supplied from Sigma-Aldrich. Both cell lines were maintained in a humidified atmosphere, 5% CO_2_, at 37 °C (incubator MCO-18AC-PE, Panasonic, Kadoma, Japan).

### 4.4. MTT Proliferation Assay

We applied with some modifications the protocol of Annex C, ISO 10995-5, based on the reduction of methylthiazol tetrazolium bromide (MTT, Merck, Darmstadt, Germany) to assess the in vitro cytotoxicity of the *C. aspersum* mucus protein fraction with MW above 20 kDa on BJ and HaCaT cells [64]. The mucus fraction was tested in a concentration range 1.5–500 µg/mL. Briefly, BJ (5 × 10^4^/well) and HaCaT (2 × 10^4^/well) cells were seeded in 96-well plates and grown as a monolayer (70–80% confluence) for 24 h in a complete growth medium. Then, the cells were incubated with 0 to 500 µg/mL (corresponding to 0 to 50 μg/well) of the tested mucus fraction in a serum-free medium for 24/48 h. After the incubation, 100 μL of DMEM containing MTT was added to each well to achieve a final concentration of 0.5 mg/mL. After 3 h of incubation at 37 °C, the supernatant was aspirated, and 100 μL of dimethylsulfoxide/ethanol (1:1, *v*/*v*) to each well were added in order to dissolve the formazan crystals. The plates were shaken for 10 min at room temperature. Then, the absorption was measured at 560 nm using a microplate reader (Biosan, Riga, Latvia). The results were calculated for each tested concentration as a percentage of the non-treated control (non-treated cells). The organic solvent was used as blank. The values were presented as the mean ± standard deviation of four independent experiments (Equation (1)).

(1) $$\%Cell\;viability=\frac{\left(A405sample-A405blank\right)}{\left(A405scontrol-A405blank\right)}\times 100$$

### 4.5. Scratch Wound-Healing Assay

The scratch wound-healing assay was carried out to assess the effects of the *C. aspersum* mucus protein fraction with MW > 20 kDa on the proliferation and migration of human keratinocytes HaCaT. Cells were seeded in 24-well plates at density of 5 × 10^5^ cells/well and incubated in 1 mL DMEM medium containing 10% at 37 °C/5% CO_2_ for 24 h. When the cells formed confluent monolayers, scratches were induced in the monolayers across the diameter of the wells using a sterile 10 μL pipette tip. The culture medium in each well was aspirated and each well was washed 4 times with PBS. Then, 1 mL fresh DMEM media (without FBS and phenol red) containing 200 μg/mL of the tested mucus fraction. Culturing medium without mucus fraction was added to the untreated control. The wound closure was monitored regularly and micrograph images were taken with the Zoe Fluorescent Cell Imager (Bio-Rad, Hercules, CA, USA).

### 4.6. Chemicals and Reagents for Identification of the Bacterial Isolates and Assessment of the Antimicrobial Activity of Mucus Fraction with MW > 20 kDa

McConkey agar (#M081), Chapman agar (#M215), Brain heart infusion agar (BHI, #M211) and Cetrimide agar (#M024) were purchased from HiMedia (Mumbai, Maharashtra, India). The chemotherapeutic ciprofloxacin (Ciproflav, Warsaw Pharmaceutical Works Polfa S.A., Warsaw, Poland) was bought from a commercial pharmacy. The working solution (40 mg/L) was prepared in situ before the experiments in phosphate Dulbecco’s phosphate-buffered saline (PBS, #D8537) was purchased from Merck (Sigma-Aldrich, Steinheim, Germany). The following antibiotic disks were used for the Kirby–Bauer disk diffusion test: PEN G-penicillin (10 Units, SD028-1PK), AMC-amoxicillin/clavulanic acid (20/10 µg, AUG30C), AMP-ampicillin (10 µg, SD002-1PK), O-Oxacillin (1 µg, SD088-1PK), V-Vancomycin (5 µg, SD155-1PK), MER-Meropene1PKm (10 µg, MEM10C Oxoid ltd, Basingstoke, Hampshire, UK), CFX-Ceftriaxone (30 µg, SD065-1PK), ERM-erythromicin (15 µg, SD013-1PK), Clarithromycin (15 µg, SD192-), T-Tetracycline (30 µg, SD037-1PK), G-Gentamicin (10 µg, SD016-1PK), AMK-Amikacin (30 µg, SD035-1PK), CHL-Chloramphenicol (30 µg, SD006-1PK), R- Rifampicin (5 µg, SD030-1PK), CIP-Ciprofloxacin (5 µg, SD060-1PK), NOR-Norfloxacin (10 µg, SD057-1PK), and T/S-Trimethoprim/sulfametoxazol (1.25/23.75 µg, SD010-1PK).

The biochemical reactions were carried out using kits and reagents as follows: citrate, fermentation of lactose, maltose, mannitol, and mannose were part of the reactions in HiCarbo™ Kit. Indole (DMACA Indole Discs) and gelatin hydrolase (Nutrient gelatin) were purchased from HiMedia (Mumbai, Maharashtra, India). Oxidase (OXItest ID diagnostic strips), urease (Christensen’s Urea Agar), nitrate reduction (Nitrate Reduction Test) and coagulase (Coagulase Test (Tubes) were purchased from Millipore^®^ (Merck KGaA, Darmstadt, Germany). Hydrogen sulfide production and the gas evolution were detected on slant Kligler Iron Agar ordered from Oxoid^TM^ (Oxoid Ltd., Cheshire, UK). Vogges-Proskauer—VP test ID diagnostic strips and VP test—Reagent for Acetoin test was purchased from Mikrolatest^®^ ID (ERBA Diagnostics Mannheim GmbH, Brno, Czech Republic). Hemolysis was performed on Blood agar plates purchased from ProMedia doo, Serbia. Catalase reaction was accomplished with 3% hydrogen peroxide purchased from Chemax Pharma, Sofia, Bulgaria.

### 4.7. Isolation, Characterization and Identification of Pathogenic Bacteria from Patients with Diabetes

**Figure 4:**
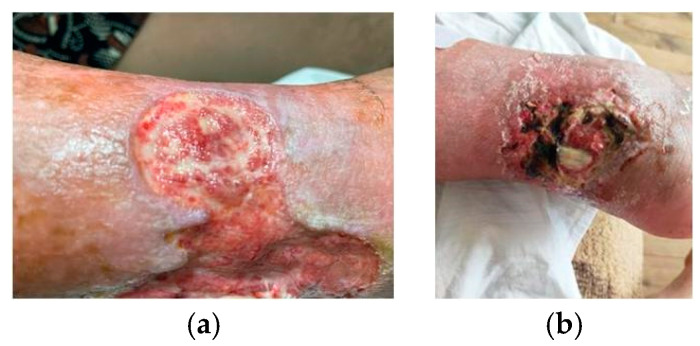
Septic wounds of patients with diabetes. Legend: (**a**) 84-year-old woman; (**b**) 54-year-old man.

The microbiological samples were provided by Dr. M. Keremedchiev. They were taken from septic wounds of two patients with diabetes—an 84-year-old woman (sample 1, Figure 4a) and a 54-year-old man (sample 2, Figure 4b). The samples were taken during the treatment of both patients with non-healing, necrotic and chronic wounds according to a newly developed surgical protocol for the treatment of acute, chronic, necrotic and non-healing wounds—the Helix Protocol. The patients were included in a 5-year, prospective clinical study for the treatment of acute, chronic, non-healing and necrotic wounds with snail mucus (from *Helix aspersa*) and herbal extracts of calendula and plantain. Sample collection protocols and patient consent conform to the principles of the Declaration of Helsinki. At the time of sampling, the patients were not on systemic antibiotic therapy. Briefly, the wound material was collected aseptically with commercial cotton swabs (#230117) after wound treatment to avoid contamination with environmental microorganisms and normal skin microflora and were subjected to microbiological analysis and antibiogram according to the established procedures. Swabs were inoculated onto the following nutrient media: McConkey agar, Chapman agar, BHI agar and Cetrimide agar under aerobic conditions at 37 °C for 24 h. For the purposes of the study, the bacterial isolates were characterized and identified using classical microbiological methods. Briefly, Gram-staining and light microscopy was done for morphological characterization. Biochemical tests (catalase, oxidase, methyl red, Vogges–Proskauer, indole, citrate, urease, nitrate reduction, hydrogen sulfide production, gas evolution, gelatin hydrolase, coagulase, hemolysis, fermentation of lactose, maltose, mannitol, mannose) were applied for preliminary identification. The identification of four pathogenic bacterial isolates was confirmed with the BD Phoenix M50 automated system (Becton Dickinson and Company—BD, Franklin Lakes, NJ, USA), including biochemical characterization and antibiotic susceptibility. For this purpose, the suspected bacterial isolates were spread on blood agar (#01011) and cultured under aerobic conditions at 37 °C for 24 h. In the next day, bacterial suspensions with optical density 0.5 McFarland were prepared in ID broth (#246001, BD, Franklin Lakes, NJ, USA). A 25 µL volume of this suspension was transferred into AST broth (#246003, BD, Franklin Lakes, NJ, USA) containing AST indicator solution (#246004, BD, Franklin Lakes, NJ, USA). The suspensions were inoculated into NMIC/ID-76 panels for Gram-negative (#448103, BD, Franklin Lakes, NJ, USA) and Gram-positive (#448796, BD, Franklin Lakes, NJ, USA) bacteria and loaded into the instrument at 35 °C for 24 ± 4 h. The data obtained were analyzed using the EpiCentre™ software (V7.45A/V6.71A) provided by BD with the BD Phoenix M50 automated system.

### 4.8. Determination of Antimicrobial Resistance with the Kirby–Bauer Disk Diffusion Test

This test was used to determine the sensitivity of the clinical isolates in this study to commercial antibiotics and chemotherapeutics impregnated on 6 mm filter paper disks [63,65,66].

Briefly, overnight cultures of the bacterial isolates were used to prepare bacterial suspensions in phosphate-buffered saline which turbidity as adjusted to a 0.5 McFarland standard using a suspension turbidity detector (Densitometer 1B, BioSan, Riga, Latvia, LV-1067). This suspension was used within 15 min of preparation. A sterile swab was dipped into the inoculum tube and rotated against the side of the tube (above the fluid level) using firm pressure, to remove excess fluid. The swab was streaked over the entire surface of the MH agar plate by rotating the plate in order to inoculate the bacterial suspension evenly. Thereafter, the appropriate antimicrobial-impregnated disks were placed on the surface of the agar using a sterile forceps and pressed to ensure complete contact. No more than 5 disks were plated on 10 mm Petri dishes. Plates were incubated under aerobic conditions at 37 °C for 18 or 24 h (for vancomycin on *S. aureus* and *E. faecalis*). The inhibition zones were measured with a ruler rounding up to the next millimeter and interpreted according to the recommendations of EUCAST [65].

### 4.9. Determination of Minimal Inhibitory Concentrations

The minimal inhibitory concentration (MIC) of the snail mucus administered alone or in combination with CIP was determined by the broth microdilution method (BMD) following ISO 20776/1 [67]. Briefly, 50 µL two-fold serial dilutions of the snail mucus starting from 25% in MHB were prepared in triplicate in 96-well plates. Ciprofloxacin (CIP) was used as positive control in concentrations ranging between 1 and 0.0156 mg/L for *S. aureus*, 0.2–0.00156 mg/L for *E. faecalis* and 4–0.03125 mg/L for *P. aeruginosa*, whereas PBS served the negative control. The culture medium was tested for lack of contamination by a parallel cultivation for the same time period. The working bacterial suspensions of the isolates were prepared for the BMD test on the following way: (1) an overnight liquid bacterial culture in MHB was diluted to 1 × 10^8^ CFU/mL (OD600, 0.5 McFarland) by using a densitometer; (2) this suspension was further diluted to 5 × 10^5^ CFU/mL as recommended by the ISO cited above; (3) 50 µL of the second bacterial suspension was added to each well of the plates with the serial dilutions of the snail mucus or the positive control. The samples were incubated at 37 °C for 24 h. the result was recorded visually, whereby the lowest mucus or drug concentration which lead to a visible inhibition of the bacterial growth was accepted as MIC according to the recommendations of EUCAST [65].

### 4.10. Checkerboard Assay

The checkerboard BMD test was used for the in vitro evaluation of combinations between the snail mucus and CIP. Both components of the combination were mixed in a 96-well plate following the classical schema of the checkerboard assay [66]. For this aim serial two-fold dilutions were prepared in a two-dimensional fashion, thereby including 42 combinations/plate. The concentrations of CIP ranged as for the BMD assay and the result was evaluated as for the determination of MIC.

The combination effects were calculated and interpreted using the fractional inhibitory concentration (FIC) methodology [68]:

(2) $$FIC(A)=\frac{{MIC}_{C}(A)}{MIC(A)}$$

(3) $$FIC(B)=\frac{{MIC}_{C}(B)}{MIC(B)},$$

where FIC means fractional inhibitory concentration; MIC_C_ is minimal inhibitory concentration in the combination; A stands for component A (snail mucus), and B is CIP.

(4) $$\sum FIC=FIC\left(A\right)+FIC(B)$$

Synergy was defined as ƩFIC ≤ 0.5, additive effect—as 0.5 < ƩFIC ≤ 1, indifference—as 0.5 < ƩFIC ≤ 4, and antagonism—as ƩFIC > 4 [68].

## 5. Conclusions

In the current study, for the first time we proved the synergistic effect between a protein fraction (MW > 20 kDa) isolated from *Cornu aspersum* mucus and the fluoroquinolone ciprofloxacin against pathogenic bacterial isolates from two patients with chronic diabetic ulcers. The proteomic analysis of the mucus fraction reveals number of proteins which possess a high homology with known antibacterial mucus proteins such as aspernin, hemocyanins, H-lectins, L-amino acid oxidase-like protein. Combination of the mucus fraction with ciprofloxacin led to significant diminishment (two- to four-fold) of the active concentrations of the antibacterial chemotherapeutic. It was demonstrated that the mucus fraction itself potentiates the wound-healing process and does not exhibit any cytotoxicity on human fibroblasts and keratinocytes. Based on the results obtained, we can conclude that the synergistic combination opens new possibilities for improving the therapeutic schemas in the treatment of DFU and is a perspective to be subjected to further pharmacological investigations of more combined treatment modalities with other antibiotics and their mode of action.

## Figures and Tables

**Table 1:** Main morphological and biochemical features of the isolates.

Characteristics	*Staphylococcus aureus*	*Enterococcus faecalis*	*Pseudomonas aeruginosa*
Gram Staining	Positive	Positive	Negative
Shape (cocci/diplococci/rods)	cocci	cocci	rods
Motility (motile/non-motile)	non-motile	non-motile	motile (unipolar)
Capsule (capsulated/non-capsulated)	non-capsulated	non-capsulated	non-capsulated
Spore (sporing/non-sporing)	non-sporing	non-sporing	non-sporing
Flagella (flagellated/non-flagellated)	non-flagellated	non-flagellated	single flagella
Catalase	positive (+)	negative (−)	positive (+)
Oxidase	negative (−)	negative (−)	positive (+)
MR (methyl red)	positive (+)	-	negative (−)
VP (Voges Proskauer)	positive (+)	positive (+)	negative (−)
OF (oxidative/fermentative)	fermentative	Fermentative	oxidative/
Indole	negative (−)	negative (−)	negative (−)
H_2_S	negative (−)	negative (−)	negative (−)
Citrate	positive (+)	negative (−)	positive (+)
Urease	positive (+)	negative (−)	negative (−)
Nitrate reduction	positive (+)	positive (+)	positive (+)
Gelatin hydrolysis	positive (+)	variable	positive (+)
Hemolysis	positive (+)-beta	variable (alfa or beta)	-
Coagulase	positive (+)	-	negative (−)
**Fermentation of**			
Lactose	positive (+)	positive (+)	negative (−)
Maltose	positive (+)	positive (+)	negative (−)
Mannitol	positive (+)	positive (+)	positive (+)

Legend: Table headings are in bold.

**Table 2:** Antibiotic susceptibility testing of the Gram-positive clinical isolates *Staphylococcus aureus* and *Enterococcus faecalis* determined by the Kirby–Bauer disk diffusion method.

No.	Antibiotic/Chemotherapeutic	Inhibition Zone [mm]	
		*Staphylococcus aureus* (Sample 1)	*Enterococcus faecalis* (Sample 2)
1.	AMC-amoxicillin/clavulanic acid (20/10 µg), 15 mm	38 (S)	54 (S)
2.	AMP-ampicillin (10 µg), 28 mm	36 (S)	48 (S)
3.	CFX-Ceftriaxone (30 µg), 13–21mm	40 (S)	44 (S)
4.	CIP-Ciprofloxacin (5 µg), 15–21 mm	28 (S)	30 (S)
5.	CHL-Chloramphenicol (30 µg), 12–18 mm	36 (S)	38 (S)
6.	Cla-Clarithromycin (15 µg), 26–32 mm	0 (R)	34 (S)
7.	T-Tetracycline (30 µg), 14–19 mm	40 (S)	40 (S)
8.	ERM-erythromicin (15 µg), 13–23 mm	0 (R)	24 (S)
9.	G-gentamicin (10 µg), 12–15 mm	14 (I)	14 (I)
10.	PEN G-penicillin (10 Units), 28–29 mm	34 (S)	40 (S)
11.	V-Vancomycin (5 µg), 9–12 mm	22 (S)	27 (S)
12.	R-Rifampin (5 µg), 16–20 mm	44 (S)	32 (S)

Legend: S—sensitive; I—intermediate; R—resistant.

**Table 3:** Antibiotic susceptibility of the Gram-negative clinical isolates *Pseudomonas aeruginosa* determined by the Kirby–Bauer disk diffusion method.

No.	Antibiotic/Chemotherapeutic	Inhibition Zone [mm]	
		*Pseudomonas aeruginosa* (Sample 1)	*Pseudomonas aeruginosa* (Sample 2)
1.	AMK-Amikacin (30 µg), 14–17 mm	18 (S)	23 (S)
2.	AMC-Amoxicillinclavulanic acid (20/10 µg), 15 mm	0 (R)	0 (R)
3.	AMP-ampicillin (10 µg), 28 mm	0 (R)	0 (R)
4.	CFX-Ceftriaxone (30 µg), 13–21mm	28 (S)	26 (S)
5.	CIP-Ciprofloxacin (5 µg), 15–21 mm	20 (I)	24 (S)
6.	G-Gentamicin (10 µg), 12–15 mm	12 (I)	10 (R)
7.	MER-Meropenem (10 µg), 27–33 mm	12(R)	18(R)
8.	NOR-Norfloxacin (10 µg), 12–17 mm	0 (R)	0 (R)
9.	O-Oxacillin (1 µg), 10–13 mm	0 (R)	0 (R)
10.	TS-Trimethoprimsulfametoxazol (1.25/23.75 µg), 9–12 mm	0 (R)	0 (R)

**Table 4:** Combination effects of snail mucus with MW > 20 kDa with CIP on isolates from patients with diabetes.

Clinical Isolates	MIC of A [mg/L]	MIC of B [mg/L]	MIC_C_ of A [mg/L]	MIC_C_ of B [mg/L]	FIC of A	FIC of B	∑FIC	Effect
*Staphylococcus aureus*	255	0.25	4	0.125	0.0157	0.5	0.5157	Additive
*Enterococcus faecalis*	>255	0.025	4	0.0125	0.0039	0.5	0.5039	Synergism
*Pseudomonas aeruginosa* (1)	>255	2	4	1	0.0039	0.5	0.5039	Synergism
*Pseudomonas aeruginosa* (2)	>255	2	4	1	0.0039	0.5	0.5039	Synergism

Legend: A—snail mucus fraction P-3; B—CIP; MIC_C_—minimal inhibitory concentration in the combination; FIC—fraction inhibitory concentration; 1—isolate 1; 2—isolate 2.

## Data Availability

All presented data will be provided if needed by the authors per e-mail.

## References

[1] Leśków A., Tarnowska M., Szczuka I., Diakowska D. (2021). The effect of biologically active compounds in the mucus of slugs *Limax maximus* and *Arion rufus* on human skin cells. Sci. Rep..

[2] Rizzi V., Gubitosa J., Fini P., Nuzzo S., Agostiano A., Cosma P. (2021). Snail slime-based gold nanoparticles: An interesting potential ingredient in cosmetics as an antioxidant, sunscreen, and tyrosinase inhibitor. J. Photochem. Photobiol. B Biol..

[3] Rashad M., Sampò S., Cataldi A., Zara S. (2023). Biological activities of gastropods secretions: Snail and slug slime. Nat. Prod. Bioprospecting.

[4] Aouji M., Rkhaila A., Bouhaddioui B., Zirari M., Harifi H., Taboz Y., Lrhorfi L.A., Bengueddour R. (2023). Chemical composition, mineral profile, anti-bacterial, and wound healing properties of snail slime of *Helix aspersa* Müller. BioMedicine.

[5] Brieva A., Philips N., Tejedor R., Guerrero A., Pivel J.P., Alonso-Lebrero J.L., Gonzalez S. (2008). Molecular basis for the regenerative properties of a secretion of the mollusk *Cryptomphalus aspersa*. Ski. Pharmacol. Physiol..

[6] Dolashki A., Velkova L., Daskalova E., Zheleva N., Topalova Y., Atanasov V., Voelter W., Dolashka P. (2020). Antimicrobial activities of different fractions from mucus of the garden snail *Cornu aspersum*. Biomedicines.

[7] McDermott M., Cerullo A.R., Parziale J., Achrak E., Sultana S., Ferd J., Samad S., Deng W., Braunschweig A.B., Holford M. (2021). Advancing discovery of snail mucins function and application. Front. Bioeng. Biotechnol..

[8] Perpelek M., Tamburaci S., Aydemir S., Tihminlioglu F., Baykara B., Karakasli A., Havitcioglu H. (2021). Bioactive snail mucus-slime extract loaded chitosan scaffolds for hard tissue regeneration: The effect of mucoadhesive and antibacterial extracts on physical characteristics and bioactivity of chitosan matrix. Biomed. Mater..

[9] Phrompanya P., Suriyaruean N., Nantarat N., Saenphet S., Tragoolpua Y., Saenphet K. (2023). Biological properties of mucus from land snails (*Lissachatina fulica*) and freshwater snails (*Pomacea canaliculata*) and histochemical study of mucous cells in their foot. PeerJ.

[10] Velkova L., Dolashki A., Petrova V., Pisareva E., Kaynarov D., Kermedchiev M., Todorova M., Dolashka P. (2024). Antibacterial Properties of Peptide and Protein Fractions from *Cornu aspersum* Mucus. Molecules.

[11] Waluga-KozŁOwska E.W.A., Jasik K., WcisŁO-Dziadecka D., Pol P., KuŹNik-Trocha K., KomosiŃSka-Vassev K., Olczyk K., Waluga M., Olczyk P., Zimmermann A. (2021). Snail mucus-a natural origin substance with potential use in medicine. Acta Pol. Pharm..

[12] Cilia G., Fratini F. (2018). Antimicrobial properties of terrestrial snail and slug mucus. J. Complement. Integr. Med..

[13] Deng T., Gao D., Song X., Zhou Z., Zhou L., Tao M., Jiang Z., Yang L., Luo L., Zhou A. (2023). A natural biological adhesive from snail mucus for wound repair. Nat. Commun..

[14] Zhu K., Zhang Z., Li G., Sun J., Gu T., Ain N.U., Zhang X., Li D. (2023). Research progress on the extraction, structure, pharmacological activities and applications of polysaccharides and proteins isolated from snail mucus. Int. J. Biol. Macromol..

[15] Cerullo A.R., McDermott M.B., Pepi L.E., Liu Z.-L., Barry D., Zhang S., Yang X., Chen X., Azadi P., Holford M. (2023). Comparative mucomic analysis of three functionally distinct *Cornu aspersum* Secretions. Nat. Commun..

[16] Pitt S.J., Graham M.A., Dedi C.G., Taylor-Harris P.M., Gunn A. (2015). Antimicrobial properties of mucus from the brown garden snail *Helix aspersa*. Br. J. Biomed. Sci..

[17] Pitt S.J., Hawthorne J.A., Garcia-Maya M., Alexandrovich A., Symonds R.C., Gunn A. (2019). Identification and characterisation of anti-*Pseudomonas aeruginosa* proteins in mucus of the brown garden snail, *Cornu aspersum*. Br. J. Biomed. Sci..

[18] Suárez L., Pereira A., Hidalgo W., Uribe N. (2021). Antibacterial, antibiofilm and anti-virulence activity of biactive fractions from mucus secretion of giant African snail *Achatina fulica* against *Staphylococcus aureus* strains. Antibiotics.

[19] Ulagesan S., Kim H.J. (2018). Antibacterial and antifungal activities of proteins extracted from seven different snails. Appl. Sci..

[20] Etim L., Aleruchi C., Obande G. (2016). Antibacterial properties of snail mucus on bacteria isolated from patients with wound infection. Br. Microbiol. Res. J..

[21] Santana W.A., Melo C.M.d., Cardoso J.C., Pereira-Filho R.N., Rabelo A.S., Reis F.P., Albuquerque-Júnior R.L.C.d. (2012). Evaluación de la Actividad Antimicrobiana y la Cicatrización Potencial de la Secreción Mucosa de Achatina fulica. Int. J. Morphol..

[22] Nuryana C.T., Haryana S.M., Wirohadidjojo Y.W., Arfian N. (2020). Achatina fulica mucous improves cell viability and increases collagen deposition in UVB-irradiated human fibroblast culture. J. Stem Cells Regen. Med..

[23] Trapella C., Rizzo R., Gallo S., Alogna A., Bortolotti D., Casciano F., Zauli G., Secchiero P., Voltan R. (2018). HelixComplex snail mucus exhibits pro-survival, proliferative and pro-migration effects on mammalian fibroblasts. Sci. Rep..

[24] López Angulo D.E., do Amaral Sobral P.J. (2016). Characterization of gelatin/chitosan scaffold blended with aloe vera and snail mucus for biomedical purpose. Int. J. Biol. Macromol..

[25] Kostadinova N., Voynikov Y., Dolashki A., Krumova E., Abrashev R., Kowalewski D., Stevanovic S., Velkova L., Velikova R., Dolashka P. (2018). Antioxidative screening of fractions from the mucus of garden snail *Cornu aspersum*. Bulg. Chem. Commun..

[26] Basavegowda N., Baek K.-H. (2022). Combination strategies of different antimicrobials: An efficient and alternative tool for pathogen inactivation. Biomedicines.

[27] Kaur I. (2016). Novel strategies to combat antimicrobial resistance. J. Infect. Dis. Ther..

[28] Bozic I., Reiter J.G., Allen B., Antal T., Chatterjee K., Shah P., Moon Y.S., Yaqubie A., Kelly N., Le D.T. (2013). Evolutionary dynamics of cancer in response to targeted combination therapy. elife.

[29] León-Buitimea A., Garza-Cárdenas C.R., Garza-Cervantes J.A., Lerma-Escalera J.A., Morones-Ramírez J.R. (2020). The demand for new antibiotics: Antimicrobial peptides, nanoparticles, and combinatorial therapies as future strategies in antibacterial agent design. Front. Microbiol..

[30] Armstrong D.G., Tan T.-W., Boulton A.J.M., Bus S.A. (2023). Diabetic foot ulcers: A review. JAMA.

[31] Frykberg R.G., Franks P.J., Edmonds M., Brantley J.N., Téot L., Wild T., Garoufalis M.G., Lee A.M., Thompson J.A., Reach G. (2020). A Multinational, Multicenter, Randomized, Double-Blinded, Placebo-Controlled Trial to Evaluate the Efficacy of Cyclical Topical Wound Oxygen (TWO_2_) Therapy in the Treatment of Chronic Diabetic Foot Ulcers: The TWO_2_ Study. Diabetes Care.

[32] Gong Q., Zhang P., Wang J., Ma J., An Y., Chen Y., Zhang B., Feng X., Li H., Chen X. (2019). Morbidity and mortality after lifestyle intervention for people with impaired glucose tolerance: 30-year results of the Da Qing Diabetes Prevention Outcome Study. Lancet Diabetes Endocrinol..

[33] Alavi A., Sibbald R.G., Mayer D., Goodman L., Botros M., Armstrong D.G., Woo K., Boeni T., Ayello E.A., Kirsner R.S. (2014). Diabetic foot ulcers: Part I. Pathophysiology and prevention. J. Am. Acad. Dermatol..

[34] Brem H., Tomic-Canic M. (2007). Cellular and molecular basis of wound healing in diabetes. J. Clin. Investig..

[35] Erem C., Hacıhasanoğlu A., Çelik Ş., Ovalı E., Ersöz H.Ö., Ukinç K., Deger O., Telatar M. (2004). Coagulation and Fibrinolysis Parameters in Type 2 Diabetic Patients with and without Diabetic Vascular Complications. Med. Princ. Pract..

[36] Galkowska H., Wojewodzka U., Olszewski W.L. (2006). Chemokines, cytokines, and growth factors in keratinocytes and dermal endothelial cells in the margin of chronic diabetic foot ulcers. Wound Repair Regen..

[37] Patel S., Srivastava S., Singh M.R., Singh D. (2019). Mechanistic insight into diabetic wounds: Pathogenesis, molecular targets and treatment strategies to pace wound healing. Biomed. Pharmacother..

[38] Yang L., Rong G.C., Wu Q.N. (2022). Diabetic foot ulcer: Challenges and future. World J. Diabetes.

[39] Mariadoss A.V.A., Sivakumar A.S., Lee C.-H., Kim S.J. (2022). Diabetes mellitus and diabetic foot ulcer: Etiology, biochemical and molecular based treatment strategies via gene and nanotherapy. Biomed. Pharmacother..

[40] Djahmi N., Messad N., Nedjai S., Moussaoui A., Mazouz D., Richard J.L., Sotto A., Lavigne J.P. (2013). Molecular epidemiology of *Staphylococcus aureus* strains isolated from inpatients with infected diabetic foot ulcers in an Algerian University Hospital. Clin. Microbiol. Infect..

[41] Lipsky B.A., Berendt A.R., Cornia P.B., Pile J.C., Peters E.J.G., Armstrong D.G., Deery H.G., Embil J.M., Joseph W.S., Karchmer A.W. (2012). 2012 Infectious Diseases Society of America Clinical Practice Guideline for the Diagnosis and Treatment of Diabetic Foot Infectionsa. Clin. Infect. Dis..

[42] Akhi M.T., Ghotaslou R., Asgharzadeh M., Varshochi M., Pirzadeh T., Memar M.Y., Bialvaei A.Z., Sofla H.S.Y., Alizadeh N. (2015). Bacterial etiology and antibiotic susceptibility pattern of diabetic foot infections in Tabriz, Iran. GMS Hyg. Infect. Control.

[43] Kwon K.T., Armstrong D.G. (2018). Microbiology and Antimicrobial Therapy for Diabetic Foot Infections. Infect. Chemother..

[44] Li X., Du Z., Tang Z., Wen Q., Cheng Q., Cui Y. (2022). Distribution and drug sensitivity of pathogenic bacteria in diabetic foot ulcer patients with necrotizing fasciitis at a diabetic foot center in China. BMC Infect. Dis..

[45] Macdonald K.E., Boeckh S., Stacey H.J., Jones J.D. (2021). The microbiology of diabetic foot infections: A meta-analysis. BMC Infect. Dis..

[46] Mamdoh H., Hassanein K.M., Eltoony L.F., Khalifa W.A., Hamed E., Alshammari T.O., Abd El-Kareem D.M., El-Mokhtar M.A. (2023). Clinical and Bacteriological Analyses of Biofilm-Forming Staphylococci Isolated from Diabetic Foot Ulcers. Infect. Drug Resist..

[47] Mariani F., Juarez G.E., Barberis C., Veiga F., Vay C., Galvan E.M. (2023). Interspecies interactions in mixed-species biofilms formed by *Enterococcus faecalis* and gram-negative bacteria isolated from polymicrobial diabetic foot ulcers. Biofouling.

[48] Shi M.-L., Quan X.-R., Tan L.-M., Zhang H.-L., Yang A.-Q. (2023). Identification and antibiotic susceptibility of microorganisms isolated from diabetic foot ulcers: A pathological aspect. Exp. Ther. Med..

[49] Thanganadar Appapalam S., Muniyan A., Vasanthi Mohan K., Panchamoorthy R. (2019). A Study on Isolation, Characterization, and Exploration of Multiantibiotic-Resistant Bacteria in the Wound Site of Diabetic Foot Ulcer Patients. Int. J. Low. Extrem. Wounds.

[50] Younes N.A., Bakri F.G. (2006). Diabetic foot infection. Saudi Med. J..

[51] Salah M., Badr G., Hetta H., Khalifa W.A., Shoreit A.A. (2022). Isolation and identification of pathogenic biofilm-forming bacteria invading diabetic wounds. Assiut Univ. J. Multidiscip. Sci. Res..

[52] Dolashka P., Atanasov D. (2013). Device for Collecting Extracts from Garden Snail.

[53] Perez-Favila A., Martinez-Fierro M.L., Rodriguez-Lazalde J.G., Cid-Baez M.A., Zamudio-Osuna M.D., Martinez-Blanco M.D., Mollinedo-Montaño F.E., Rodriguez-Sanchez I.P., Castañeda-Miranda R., Garza-Veloz I. (2019). Current Therapeutic Strategies in Diabetic Foot Ulcers. Medicina.

[54] Singh S.K., Gupta B. (2017). Choices and Challenges of Antibiotics Therapy in Diabetic Foot Infection. Indian J. Endocrinol. Metab..

[55] Filius P.M.G., Gyssens I.C. (2002). Impact of Increasing Antimicrobial Resistance on Wound Management. Am. J. Clin. Dermatol..

[56] LeBel M. (1988). Ciprofloxacin: Chemistry, Mechanism of Action, Resistance, Antimicrobial Spectrum, Pharmacokinetics, Clinical Trials, and Adverse Reactions. Pharmacother. J. Hum. Pharmacol. Drug Ther..

[57] Campbell K.B., Tisdale J.E. (2022). Chapter 10—Antimicrobial agents and torsades de pointes. Torsades de Pointes.

[58] Iguchi S.M.M., Aikawa T., Matsumoto J.J. (1982). Antibacterial activity of snail mucus mucin. Comp. Biochem. Physiol. Part A Physiol..

[59] Abimbola Okeniyi F., Oghenebrorhie Mavis O., Oyewale Olawoye S., Adekunle Animashahun R., Gbemisola Adeyonu A. (2022). Antimicrobial potentials of mucus mucin from different species of giant African land snails on some typed culture pathogenic bacteria. Asian J. Agric. Biol..

[60] El-Zawawy N.A., Mona M.M. (2021). Antimicrobial efficacy of Egyptian *Eremina desertorum* and *Helix aspersa* snail mucus with a novel approach to their anti-inflammatory and wound healing potencies. Sci. Rep..

[61] Bradford M.M. (1976). A rapid and sensitive method for the quantitation of microgram quantities of protein utilizing the principle of protein-dye binding. Anal. Biochem..

[62] Laemmli U.K. (1970). Cleavage of structural proteins during the assembly of the head of bacteriophage T_4_. Nature.

[63] Lincz L.F., Scorgie F.E., Garg M.B., Gilbert J., Sakoff J.A. (2020). A simplified method to calculate telomere length from Southern blot images of terminal restriction fragment lengths. Biotechniques.

[64] (2019). Biological Evaluation of Medical Devices. Part 5: Tests for In Vitro Cytotoxicity.

[65] EUCAST Clinical Breakpoints—Breakpoints and Guidance. https://www.eucast.org/clinical_breakpoints.

[66] Orhan G., Bayram A., Zer Y., Balci I. (2005). Synergy tests by E test and checkerboard methods of antimicrobial combinations against *Brucella melitensis*. J. Clin. Microbiol..

[67] (2019). Susceptibility Testing of INFECTIOUS Agents and Evaluation of Performance of Antimicrobial Susceptibility Test Devices.

[68] Jenkins S.G., Schuetz A.N. (2012). Current Concepts in Laboratory Testing to Guide Antimicrobial Therapy. Mayo Clin. Proc..

